# Elevated miR-301a expression indicates a poor prognosis for breast cancer patients

**DOI:** 10.1038/s41598-018-20680-y

**Published:** 2018-02-02

**Authors:** Jin-Zhou Zheng, Yan-Ni Huang, Ling Yao, Yi-Rong Liu, Sheng Liu, Xin Hu, Zhe-Bin Liu, Zhi-Min Shao

**Affiliations:** 1 Key Laboratory of Breast Cancer in Shanghai, Department of Breast Surgery, Fudan University Shanghai Cancer Center, 270 Dong’an Road, Shanghai, 200032 China; 2 Department of Oncology, Shanghai Medical College, Fudan University, 138 Yixueyuan Rd, Shanghai, 200032 China; 3 Department of General Surgery (Pudong Branch), Longhua Hospital Affiliated to Shanghai University of Traditional Chinese Medicine, 725 Wanping South Road, Shanghai, 200032 China; 4 Department of Surgery and Pharmacology Laboratory of Traditional Chinese Medicine, Long Hua Hospital, Shanghai University of Traditional Chinese Medicine, 725 South Wanping Road, Shanghai, 200032 China

## Abstract

Although microRNA-301a (miR-301a) has been reported to function as an oncogene in many human cancers, there are limited data regarding miR-301a and breast tumours. In this study, we first detected the expression of miR-301a using an *in situ* hybridization (ISH) -based classification system in 380 samples of BC tissue, including both non-TNBC (triple-negative breast cancer) and TNBC specimens. Our results suggest that analysing miR-301a expression in breast tissue biopsy specimens at the time of diagnosis could have the potential to identify patients who might be candidates for active surveillance. We validated our results that higher expression of miR-301a is associated with a decreased OS in independent public breast cancer databases, such as TCGA and METABRIC, using the online webtool Kaplan-Meier Plotter, which provided additional powerful evidence to confirm the prognostic value of miR-301a. MiR-301a may serve as a potential therapeutic target for patients with breast cancer. According to our results, miR-301a should be considered, and novel therapeutic options are needed to target this aggressive miR-301a-positive type of breast cancer to reduce recurrence and the mortality rate.

## Introduction

Breast cancer (BC) remains the leading cause of death among women diagnosed with cancer worldwide; Shanghai has a breast cancer incidence that surpasses all other cancer registries in China^1^. Although a rapidly growing number of treatment modalities have remarkably improved patient survival, approximately 20–30% of early-stage breast cancer cases will eventually experience recurrence and develop distant metastasis^2^. Individual biomarkers for BC, which can provide more specific information predicting patients’ prognosis, are quite necessary due to the molecular heterogeneity of BC. To date, biomarkers such as molecular subtypes according to the gene expression profiles (luminal A, luminal B, human epidermal growth factor receptor 2 [HER2] overexpression and basal-like) and commercialized kits such as Oncotype DX test, PAM50 assay, and MammaPrint test, have already been and applied in clinical treatments and prognosis prediction^3,4^. Meanwhile, numerous new biomarkers are being researched and are still urgently needed to help optimize personalized treatment in BC management.

MicroRNAs (miRNAs) are a series of single-stranded, none-protein-coding RNAs with a length of 18–22 nucleotides. More than 28,000 miRNAs from 223 species have been recorded in miRBase release 21.0 (http://www.mirbase.org) since these post-transcriptional regulators were discovered in C. elegans two decades ago^5^. Increasing evidence indicates that miRNAs play critical roles in various important cellular biological activities, such as growth, apoptosis, development and tumourigenesis through a very complicated post-transcriptional gene expression regulation network^6,7^.

Links between the dysregulation of miRNA expression and human diseases such as cancers, neurodegenerative diseases and immune-related diseases are commonly reported^8^. Previous studies have validated that miRNAs play a dual role in human cancers, being divided into oncogenic miRs and tumour suppressor miRs^9^. For example, the most-studied specific miRNA, miR-21, is described to be a common oncomiR that is overexpressed in many human cancers including BC^10,11^. In addition, miR-21 may also act as a tumour suppressor, which inhibits tumour development^12^.

In addition, due to the characteristic that miRNAs generally can be well-preserved in FFPE tissues, body fluids and other types of specimen^13,14^, they can provide clinically relevant information about cancer patients. Therefore, miRNA expression signatures have been identified as promising biomarkers with diagnostic and prognostic values in cancer treatment^15^. Accumulating evidence indicates that miR-301a acts as an oncomiR, and it has been related to tumour progression in several types of cancer. For example, miR-301a has been described as a potential marker for metastasis in prostate cancer, and its high expression was associated with an increased risk of recurrence^16^. Overexpressed miR-301a has also been observed in gastric cancer as well^17^. It has also been demonstrated to promote cancer cell progression and proliferation in other kinds of cancer, such as pancreatic cancer, colourectal cancer, melanoma and non-small-cell lung cancer^18–21^. In BC, studies have revealed that miR-301a expression was also elevated markedly and correlated with a poor prognosis in the specific subtype TNBC^22,23^. Despite these previous studies, the validation of miR-301a expression level and its precise role in a whole BC population is still needed. Therefore, in this study, we aimed to profile its exact expression level and to identify the potential prognostic role of miR-301a as a biomarker for BC.

## Results

### Patients’ characteristics and miR-301a expression pattern

**Figure 1:**
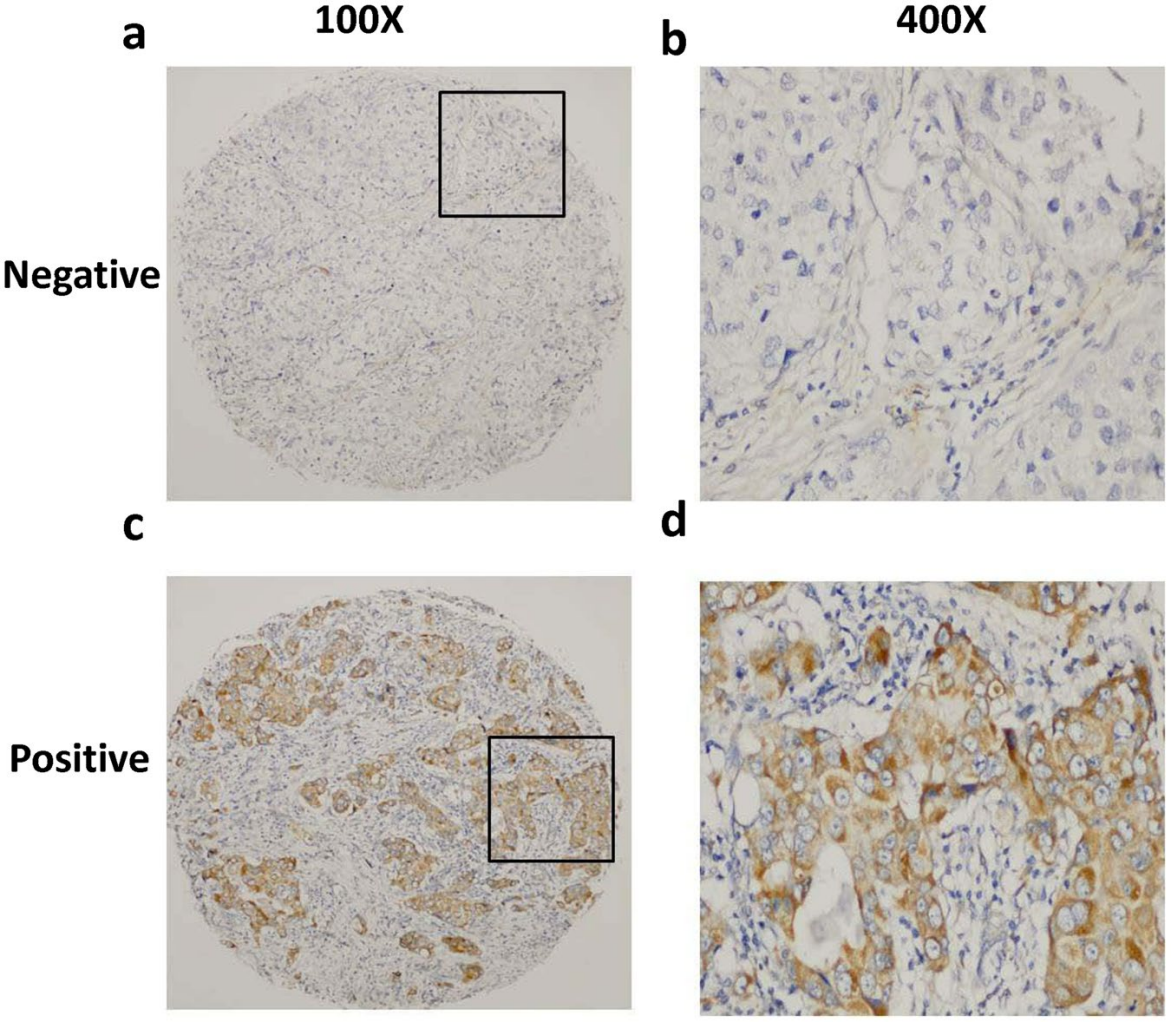
Identification of miR-301a in breast tumours by *in situ* hybridization (ISH). Representative staining of miR-301a in low-magnification (100×) and high-magnification (400×) images. (**a**,**b**) Negative miR-301a staining (grade ≤ 2); (**c**,**d**) positive miR-301a staining (grade = 3).

The relative expression of miR-301a was determined in 380 BC tissue samples. As mentioned in the staining evaluation, all patients were divided into two groups: high miR-301a expression levels (=3) were detected in 141 (37.1%) of 380 tumours, and low miR-301a expression levels (≤2) were detected in the remaining 239 (62.9%) tumours. All tumours in the study population were diagnosed as invasive carcinomas. Representative immunostaining images of miR-301a expression are presented in Fig. 1. The average age of all patients was 51.6 years (SD 9.9, median age 50, IQR 45–58 years) at the time of diagnosis. Of those known, 74.7% and 40.8% of patients had grades I/II and III cancer; 28.4%, 50.0%, and 21.6% of patients had stages I, II, and III cancers at diagnosis; and 55.0%, 65.0%, and 42.9% of patients were diagnosed with ER-positive, PR-positive, and HER2-positive cancers, respectively. Of those known with completed tumour expression, 34.2% had a triple-negative phenotype. Then, the correlations of miR-301a expression with clinicopathological characteristics of patients were statistically analysed and are presented in Table 1. The correlations between miR-301a expression and other parameters were analysed using chi-square tests and are also presented in Table 1. There was a slightly higher chance for the postmenopausal patients to have positive miR-301a expression than the premenopausal group (21.3% vs. 15.8%, *P* = 0.014). There were no significant correlations between miR-301a expression and variables such as age, tumour size, tumour grade, lymph node status, cancer stage, hormone receptors, HER2 or molecular subtype (*P* > 0.05).

**Table 1:** Clinicopathological variables and expression of miR-301a in the study cohort.

Variables	Patient Number(%)	miR-301a expression		*P*^a^ Value
		Negative N (%)	Positive N (%)	
Total	380	239(62.9)	141(37.1)	
Age(years)	380	51.0 ± 9.9	52.7 ± 9.8	0.914
Menopausal status				0.014
Pre	193(50.8)	133(35.0)	60(15.8)	
Post	187(49.2)	106(27.9)	81(21.3)	
TNM stage				0.079
I	108(28.4)	63(16.6)	45(11.8)	
II	190(50.0)	130(34.2)	60(15.8)	
III	82(21.6)	46(12.1)	36(9.5)	
Grade				0.189
1 or 2	284(74.7)	184(48.4)	100(26.3)	
3	96(25.3)	55(14.5)	41(10.8)	
Tumour size				0.081
≤2 cm	179(47.1)	112(29.5)	67(17.6)	
>2, ≤5 cm	183(48.2)	120(31.6)	63(16.6)	
>5 cm	18(4.7)	7(1.8)	11(2.9)	
Lymph node status				0.950
Negative	211(55.5)	133(35.0)	78(20.5)	
Positive	169(44.5)	99(26.1)	63(16.6)	
ER status				0.169
Negative	209(55.0)	125(32.9)	84(22.1)	
Positive	171(45.0)	114(30.0)	57(15.0)	
PR status				0.063
Negative	247(65.0)	147(38.7)	100(26.3)	
Positive	133(35.0)	92(24.2)	41(10.8)	
HER-2 status				0.911
Negative	217(57.1)	137(36.1)	80(21.1)	
Positive	163(42.9)	102(26.8)	61(16.1)	
Molecular subtype^b^				0.159
Luminal like	176(46.3)	119(31.3)	57(15.0)	
HER-2 overexpression	74(19.5)	46(12.1)	28(7.4)	
Triple negative	130(34.2)	74(19.5)	56(14.7)	

ER, estrogen receptor; PR, progesterone receptor; HER-2, human epidermal growth factor receptor 2.

^a^Based on Pearson χ^2^ test, Fisher’s exact test was used when needed.

^b^Definition of molecular subtypes: luminal like (ER and/or PR positive), HER-2 overexpression (ER and PR negative, HER-2 positive), and triple negative (ER negative, PR negative and HER-2 negative).

### MiR-301a expression is associated with clinical survival outcomes in BC patients

We analysed the relationship between miR-301a expression and prognosis. By the end of the study, a total of 83 (21.8%) of the 380 patients experienced disease recurrence or metastasis and 40 (10.5%) died of breast cancer. Among the 83 patients who experienced disease recurrence or metastasis, 61 patients (73.5%) were miR-301a positive and 22 (26.5%) were negative. The median time to relapse or death was 33.1 or 40.21 months, respectively. The DFS and OS at 5 years were 81% and 91%, and the DFS and OS at 10 years were 73% and 87%, respectively.

**Figure 2:**
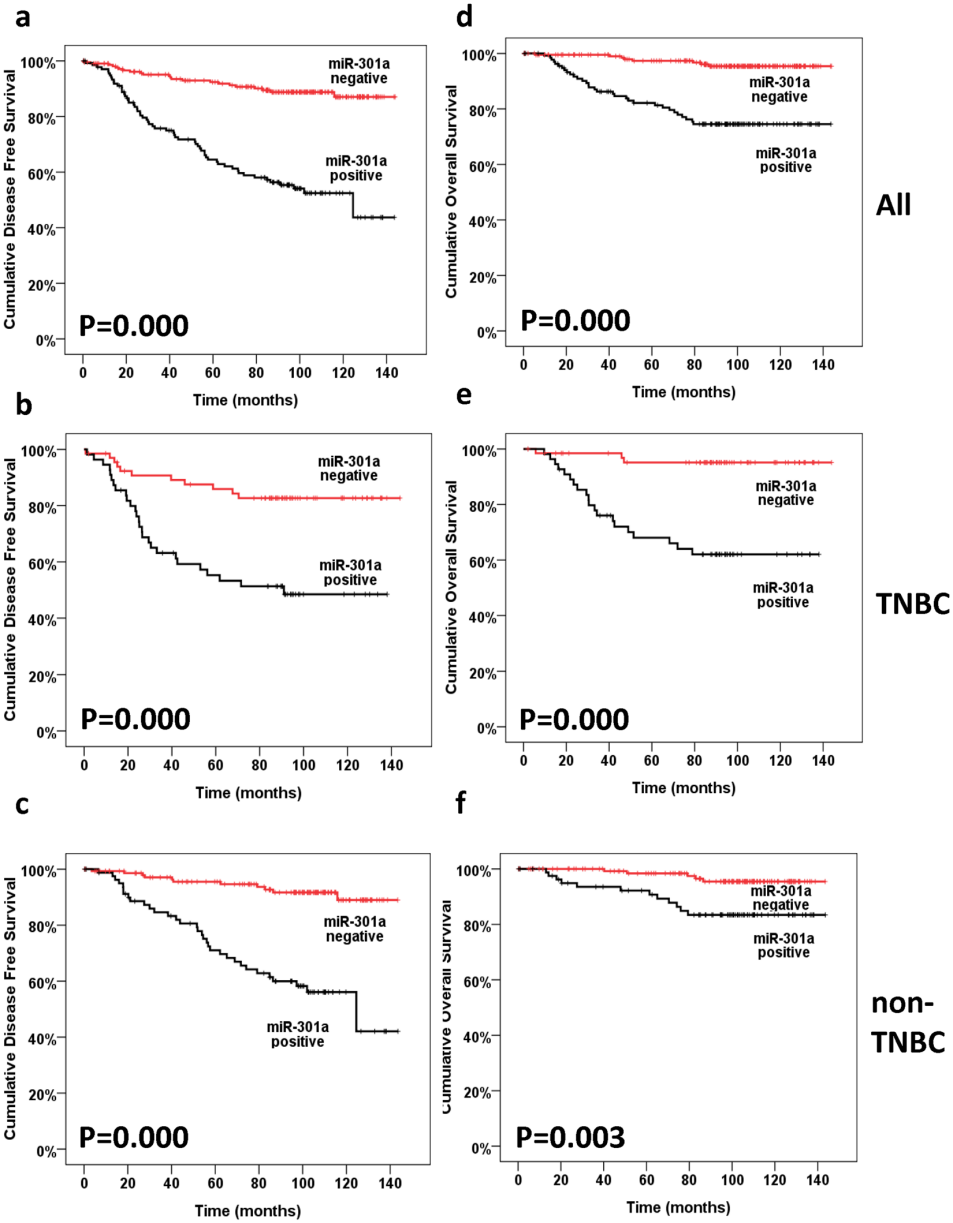
Kaplan-Meier survival curves of BC patients in our study. Kaplan-Meier curves showing the relationships between miR-301a expression and DFS (**a**–**c**) and OS (**d**–**f**) in patients with breast cancer (**a** and **d)**, all breast cancer patients; (**b** and **e)**, triple-negative breast cancer patients; (**c** and **f**), non-triple-negative breast cancer patients). Patients expressing high levels of miR-301a had a significantly shorter DFS and OS.

Kaplan-Meier analyses, timetables and log-rank tests were used to calculate the effect of miR-301a expression on patient survival and 5-year survival. The 5-year DFS of the high-miR-301a expression group (60.5%) was significantly reduced compared to that of low-miR-301a expression group (90.7%; *P* = 0.000; Fig. 2a). Moreover, the 5-year OS of the high-miR-301a expression group (74.5%) was significantly reduced compared to that of low-miR-301a expression group (96.1%; *P* = 0.000; Fig. 2d). These results indicated that downregulation of miR-301a might be correlated with better survival of BC patients.

Upon stratification of patients according to molecular subtype, we performed a subgroup analysis for the TNBC (34.2%, 130/380) and non-TNBC (65.8%, 250/380) subgroups. The expression level of miR-301a was prognostic in not only the TNBC subgroup but also the non-TNBC subgroup. Specifically, in the TNBC subgroup, there was a 30.6% difference in DFS at 5 years between patients with high-miR-301a expression tumours versus low-miR-301a expression tumours (55.3% vs. 85.9%, respectively). The difference in OS at 5 years between high-miR-301a and low-miR-301a TNBC tumours was 27.1% (68% vs. 95.1%, respectively). In the non-TNBC subgroup, the 5-year DFS and OS of patients whose tumours expressed high levels of miR-301a were only 56.1% and 83.4%, whereas the DFS and OS of those with low levels of miR-301a expression were 91.7% and 95.4%, respectively. Kaplan-Meier survival estimates demonstrated that patients with high expression levels of miR-301a had shorter DFS and OS compared to those with low expression levels of miR-301a in both the TNBC (*P* = 0.000 & *P* = 0.000, respectively; Fig. 2b and e) and non-TNBC (P = 0.000 & P = 0.003, respectively; Fig. 2d and f) subgroups.

**Figure 3:**
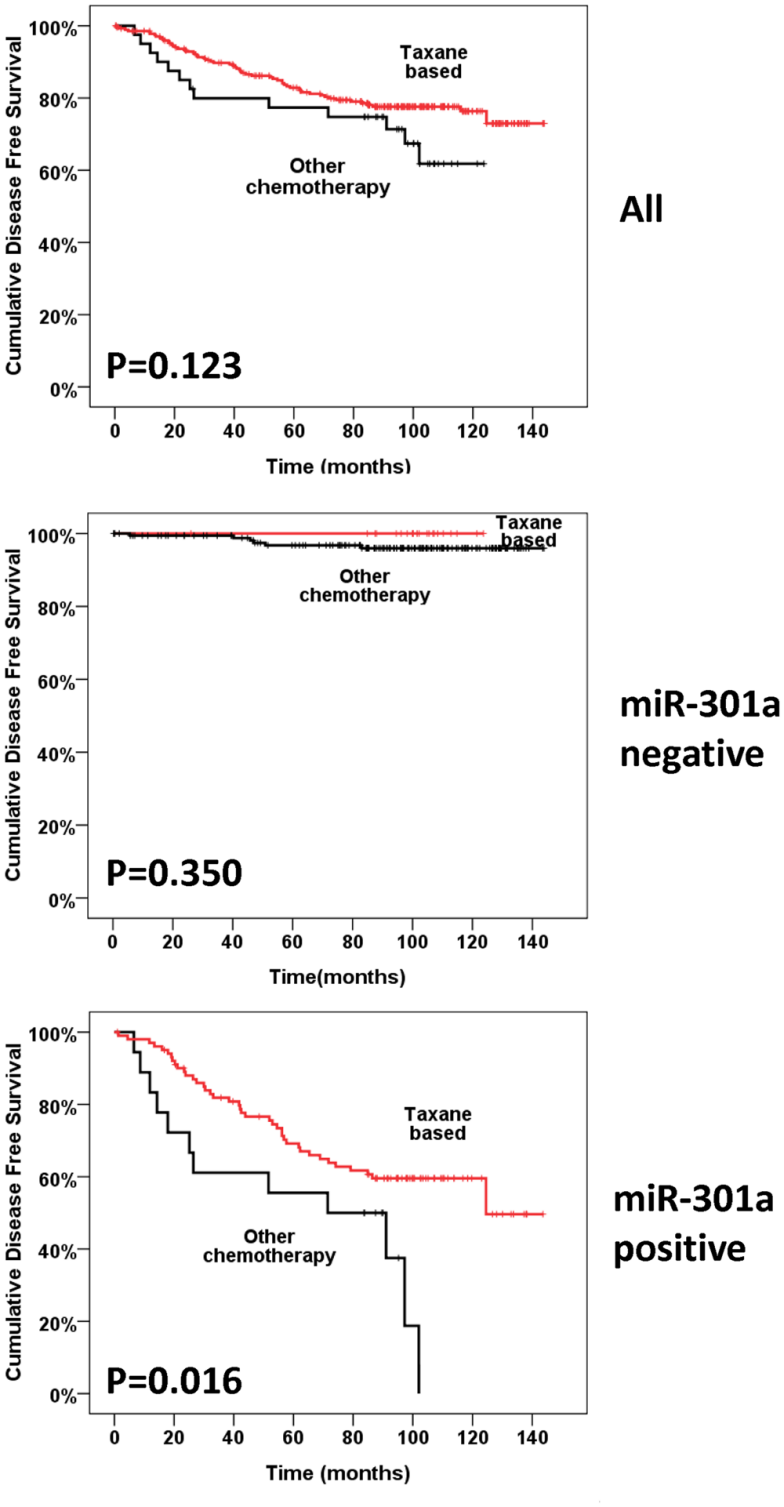
Kaplan-Meier analyses of DFS in patients of breast cancer who received taxane-based chemotherapy or other chemotherapy. Cumulative DFS curves of breast cancer compared patients who received taxane-based chemotherapy with patients who received other chemotherapy in the overall population (**a**), subgroup with miR-301a negative expression status (**b**) and subgroup with miR-301a positive expression status (**c**).

In our cohort, 341 of the 380 (89.7%) patients had received adjuvant chemotherapy after surgery. Of these, 42 patients had received Taxane-based chemotherapy and 299 patients had received chemotherapy without taxane (other chemotherapy such as CEF (anthracycline plus cyclophosphamide or anthracycline plus cyclophosphamide and 5-florouracil), CMF (cyclophosphamide plus methotrexate and 5-florouracil or vinorelbine). As shown in Fig. 3a, there was no statistically significant difference in DFS between patients who received taxane-based chemotherapy and those who received other chemotherapy for the overall population. We next investigated the response to chemotherapy according to expression status of miR-301a. In patients with positive miR-301a expression, patients who had received taxane-based chemotherapy had a better clinical outcome than those who had received other chemotherapy (P = 0.016, Fig. 3c). However, a better response to taxane was not observed among miR-301a negative patients (P = 0.350, Fig. 3b).

### Univariate and multivariate determination of prognostic factors in BC patients

Next, univariate analyses were performed to evaluate the expression of miR-301a and other clinicopathologic features on the prognosis of BC patients. As shown in Table 2, high miR-301a expression along with middle tumour size (>2,≤5 cm), lymph node metastasis, high clinical stage and molecular subtype (HER-2 overexpresstion or TNBC) were responsible for poor DFS in BC patients. All variables except age, menopausal status, and hormone receptor status were prognostic predictors of OS (Table 3).

**Table 2:** Univariate and multivariate analysis of factors for disease-free survival (DFS).

	Univariate analysis		Multivariate analysis	
	HR (95% CI)	P value	HR (95% CI)	P value
Years	1.009 (0.987–1.032)	0.408		
Menopausal status				
Pre	1			
Post	0.675 (0.437–1.043)	0.675		
Grade				
1 or 2	1			
3	1.201 (0.954–1.511)	0.119		
Tumour size				
≤2 cm	1		1	
>2, ≤5 cm	0.404 (0.180–0.908)	0.028	0.608 (0.266–1.391)	0.239
>5 cm	0.465 (0.208–1.041)	0.063	0.671 (0.294–1.528)	0.342
Lymph node status				
Negative	1		1	
Positive	0.540 (0.350–0.833)	0.005	0.462 (0.295–0.725)	0.001
TNM stage				
I	1			
II	0.370 (0.203–0.675)	0.001		
III	0.500 (0.304–0.821)	0.006		
ER status				
Negative	1			
Positive	1.348 (0.868–2.092)	1.184		
PR status				
Negative	1			
Positive	1.571 (0.965–2.559)	0.069		
HER-2 status				
Negative	1			
Positive	1.463 (0.924–2.316)	0.105		
Molecular subtype				
Luminal like	1		1	
HER-2 overexpression	0.614 (0.387–0.977)	0.039	0.606 (0.375–0.981)	0.042
Triple negative	0.498 (0.255–0.975)	0.042	0.437 (0.222–0.860)	0.017
MiR-301a				
Negative	1		1	
Positive	0.193 (0.118–0.314)	0.000	0.193 (0.118–0.316)	0.000

ER, estrogen receptor; PR, progesterone receptor; HER-2, human epidermal growth factor receptor 2; CI, confidence interval.

**Table 3:** Univariate and multivariate analysis of factors for overall survival (OS).

	Univariate analysis		Multivariate analysis	
	HR (95% CI)	P value	HR (95% CI)	P value
Years	1.039 (1.008–1.070)	0.014	1.035 (0.994–1.077)	0.095
Menopausal status				
Pre	1		1	
Post	0.474 (0.247–0.907)	0.024	0.965 (0.406–2.298)	0.937
Grade				
1 or 2	1		1	
3	0.425 (0.228–0.793)	0.007	0.433 (0.212–0.884)	0.022
Tumour size				
≤2 cm	1		1	
>2, ≤5 cm	0.176 (0.068–0.455)	0.000	0.279 (0.100–0.775)	0.014
> 5 cm	0.237 (0.094–0.596)	0.002	0.366 (0.133–1.004)	0.051
Lymph node status				
Negative	1		1	
Positive	0.295 (0.150–0.581)	0.000	0.254 (0.124–0.521)	0.000
TNM stage				
I	1			
II	0.143 (0.058–0.353)	0.000		
III	0.158 (0.077–0.324)	0.000		
ER status				
Negative	1			
Positive	1.269 (0.674–2.389)	0.460		
PR status				
Negative	1			
Positive	1.916 (0.912–4.025)	0.086		
HER-2 status				
Negative	1			
Positive	2.391 (1.138–5.023)	0.021		
Molecular subtype				
Luminal like	1		1	
HER-2 overexpression	0.507 (0.268–0.959)	0.037	0.709 (0.331–1.517)	0.375
Triple negative	0.078 (0.010–0.575)	0.012	0.061 (0.008–0.458)	0.007
MiR-301a				
Negative	1		1	
Positive	0.148 (0.068–0.322)	0.000	0.162 (0.073–0.361)	0.000

ER, estrogen receptor; PR, progesterone receptor; HER-2, human epidermal growth factor receptor 2; CI, confidence interval.

Furthermore, multivariate analyses were performed to evaluate the clinicopathological features that were significant in the univariate analyses. Since TNM stage had overlap with tumour size and lymph node, and HER-2 status had overlap with molecular subtype, thus we didn’t incorporate TNM stage and HER-2 status into multivariate analyses to avoid study bias. It was shown that a high level of miR-301a expression was an independent molecular biomarker for predicting DFS (HR: 0.193, 95% CI: 0.118–0.316, *P* = 0.000) and OS (HR: 0.162, 95% CI: 0.073–0.361, *P* = 0.000) in BC patients (Tables 2 & 3). In addition, in the multivariate analyses for DFS and OS, lymph node metastasis and HER-2/TNBC molecular subtype were independent molecular biomarkers for DFS (Table 2), while high grade, middle tumour size (>2, ≤5 cm), lymph node metastasis, and TNBC subtype were significantly and independently associated with OS (Table 3).

### External validation via online webtool

**Figure 4:**
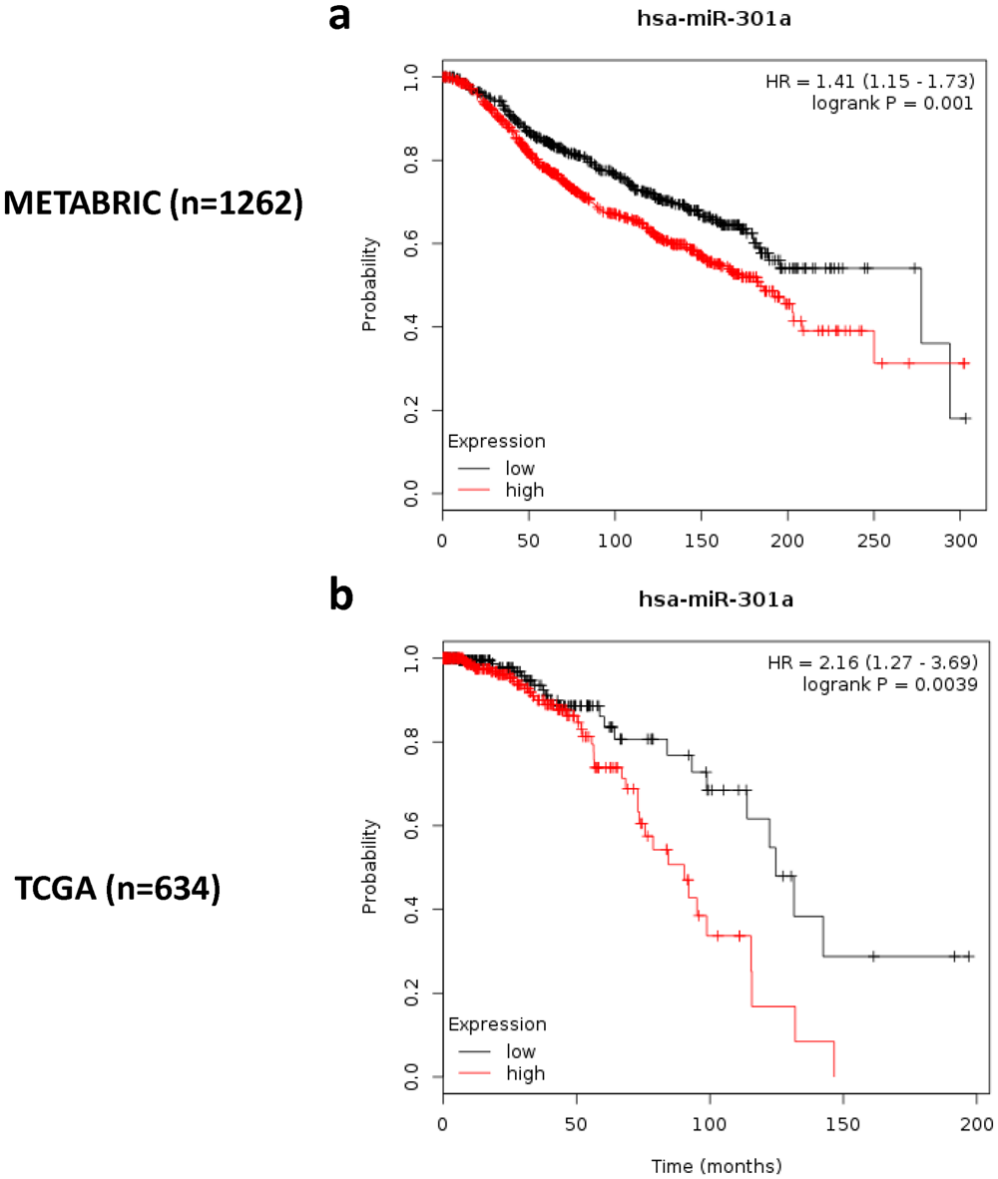
Kaplan-Meier survival curves of BC patients in public databases. Kaplan-Meier curves with log-rank analyses for patients with negative miR‐301a expression versus those with positive miR‐301a expression in the METABRIC (**a**, n = 1262) and TCGA databases (**b**, n = 634). The OS of BC patients with high miR-301a expression was significantly higher than that of those patients with low miR-301 expression.

To test the clinical relevance of the dysregulated miR-301a in BC, the data were subjected to a survival analysis based on publicly available datasets. Kaplan-Meier Plotter is an integrated online bioinformatics tool used to validate the prognostic relevance of miRNAs in breast cancer. It was set up by searching The Cancer Genome Atlas (TCGA), the Molecular Taxonomy of Breast Cancer International Consortium (METABRIC) and PubMed repositories to identify datasets with published miRNA expression and clinical data. The complete analysis tool can be accessed online at www.kmplot.com/mirpower. Data from the Kaplan-Meier Plotter database showed that patients with high expression levels of miR-301a showed a significantly worse OS time than those with low expression levels of miR-301a in both the METABRIC and TCGA databases (Fig. 4a and b, *P* = 0.001 & *P* = 0.0039, respectively).

## Discussion

A better understanding of the molecular mechanisms involved in BC initiation and progression and the proper validation of prognostic biomarkers and therapeutic targets for BC therapy have been important clinical issues in breast cancer research. MiRNAs, a class of small non-coding RNAs (~22 nt), play various roles in cancer, are still under investigation and have emerged as a new class of promising cancer biomarkers^7,9^. MiRNAs are involved in the regulation of gene expression by inducing mRNA degradation or repressing mRNA translation^24^. Recently, the correlations of dysregulated miRNAs in human BCs are increasingly reported. However, there have been a limited number of studies on the potential of miRNAs to be used for prognostic biomarkers and therapeutic molecular targets in BC. Here, the focus is on miR-301. Intronic miR-301a, which is one of two mature forms of miR-301 (miR-301a and miR-301b), and its host gene, SKA2 (spindle and kinetochore-associated protein 2), are located on chromosome 17q22–23 in the human genome^25^. MiR-301a has been reported to be dysregulated not only in breast cancer^22,23,26^ but also in a variety of malignant tumours, including prostate, gastric, pancreatic, lung, blood, colourectal, and melanoma^17–19,21,25,27–29^. As most studies have concluded, miRNA-301a was identified as an oncogenic miR, which played an important role in activating tumour cell invasion/migration, promoting cell proliferation, inhibiting apoptosis and enhancing chemosensitivity both *in vitro* and *in vivo*. This suggested that miRNA-301a may act as an oncogene in cancer and might provide helpful therapeutic strategies in clinical application.

Numerous studies have highlighted the roles of miRNAs as novel diagnostic and prognostic indicators and as potential therapeutic targets for breast cancer^30^. However, there are limited data regarding miR-301a and breast tumours; we found several papers focusing on basic research and only one on clinical research correlating miR-301a with TNBC. In the clinical study, the expression level of miR-301a was examined by quantitative reverse transcription polymerase chain reaction (qRT-PCR) in 118 pairs of TNBC tumours^22^. High miR-301a expression was significantly associated with larger tumour size, lymph node metastasis and poor OS for patients with TNBC. However, the study has revealed that miR-301a expression was correlated with a poor prognosis only in the specific subtype TNBC^22^. Here, our study first validated miR-301a expression level and detected the expression of miR-301a and its precise role in 380 BC tissues from both TNBC and non-TNBC patients and analysed its clinicopathological and prognostic significance. The experiments in our study were done by ISH on the FFPE tissue, unlike qRT-PCR, ISH could not only semi-quantitative the expression of target gene, but also roughly localize the cell type of the expressed miR-301a on FFPE block section. Several of the dysregulated miRNAs have been previously published by our study team in breast cancer^31,32^. Consistent with findings in previous studies, our results indicated that the status of miR-301a expression in tissues was significantly correlated with poor DFS and OS in breast cancer patients. Our study demonstrated that miR-301a played a role in not only the TNBC subgroup but also non-TNBC patients. More importantly, both the univariate and multivariate survival analyses demonstrated that high miR-301a expression was correlated with shorter DFS and OS in BC patients, which was also consistent with the prognostic significance of miR-301 in other human malignancies, such as gastric cancer^17^ and melanoma^18^. Further analysis showed that in miR-301a positive subgroup, TNBC had a worse OS when compared with non-TNBC patients, in the meantime, the difference was not significant when compared TNBC with non-TNBC patients in miR-301a negative subgroup (data not shown). MiR-301a has been demonstrated to have prognostic value in both TNBC and non-TNBC patients, our data added weight to the theory that TNBC patients with positive expression of miR-301a would have worse prognosis when compared with non-TNBC patients.

To further understand the potential prognostic value of miR-301a on the clinical outcome of breast cancer, the online webtool Kaplan-Meier Plotter was used to determine the prognostic value of the target gene in public breast cancer databases^33^. Altogether, 2178 cases from four independent datasets, including 2 of the most well recognized large databases METABRIC and TCGA, were integrated into the system and included the expression of 1052 distinct human miRNAs. We validated our results that higher expression of miR-301a is associated with decreased OS in independent overall public breast cancer databases such as TCGA and METABRIC as well, which provided another powerful piece of evidence to confirm the prognostic value of miR-301a. In addition, the webtool allows for the selection of patients, which can be filtered by receptors status, lymph node involvement, histological grade, and treatment types. We tried to perform subgroup analyses, and the data indicated that miR-301a is associated with a poor overall survival in the lymph node-positive subgroup and has a trend to be significantly related to a decreased OS in the TNBC subgroup based on data from the TCGA database (data not shown).

The precise mechanism(s) and targets of miR-301a in breast cancer are not fully elucidated. Based on a study by Shi *et al*., a novel oncogenic role for miR-301 through the regulation of key signalling pathways involving PTEN, FOXF2, and Col2A1 was identified, and miR-301 overexpression was associated with an increased risk of distant relapse and resistance to tamoxifen^26^. We learned from data reported by Ma *et al*. that miR-301a enforced its oncogenic function in breast cancer via inactivating PTEN, consequently activating the Wnt/β-catenin pathway^23^. Our results were consistent with the basic studies showing that overexpression of miR-301a was more aggressive and played a role in breast cancer progression and metastasis. Since most of our patients accepted tamoxifen, we were unable to test if expression of miR-301a was relevant to endocrine resistance. Taxane is an anticancer drug which is a microtubule-stabilizing compound that inhibits microtubule depolymerization within the cell^34^, and the benefit of taxane in breast cancer is supported by several studies. Previous research has shown that miRNAs are involved in chemoresistance by impacting target genes or signal pathways^35–37^, which was consistent with our results. You can tell by the intuitionistic Kaplan-Meier curves in Fig. 3 that even patients with positive miR-301a who received taxane-based chemotherapy had a significantly worse disease free survival rate when compared with those with negative miR-301a who received other chemotherapy. However, in our study, the patients with positive miR-301a status derived more benefit from taxane based chemotherapy than those with negative miR-301a status. As we all know, taxane based chemotherapy are more effective than other chemotherapeutic regimens including CEF and CMF. Thus, in our paper, what we want to tell was that although miR-301a may resistant to chemotherapy, if you have to choose, taxane-based chemotherapy would be a better choice as it had a better clinic outcome compared with other chemotherapy. Based on literature search, miR-301a specifically has not been published to have any relationship with chemoresistance. We are working on the signal pathway and chemosensitivity of miR-301a using cell lines and samples from a pre-designed neoadjuvant chemotherapy project. Data will be published in another paper. More clinic and basic research is required to elucidate the mechanism of sensitivity and/or resistance to taxane-based chemotherapy in the miR-301a-positive patients in our study population.

The strength of this study is the completeness and high degree of accuracy of the data collection. The limitations of the study are listed as follows. First, as the number of patients in this study is smaller, a larger case population is needed to confirm the prognostic value of miR-301a expression in BC. Second, we had a relatively high mortality rate, which was likely due to the specific time period of study, where the use of targeted therapy such as trastuzumab and the use of neoadjuvant chemotherapy were still not widely available. Even when trastuzumab became available, the use was limited. Third, of those studies available online, our cohort had a smaller positive miR-301a rate compared with the data published in breast cancer, gastric cancer and melanoma (37.1% vs. 58.5%, 60.9%, and 60.9%, respectively)^17,18,22^. The subjects we investigated were from 10 years ago, and formalin-fixed, paraffin-embedded (FFPE) tissues display degradation of nucleic acids compared to fresh samples. The other reasons may be due to different tissues that we tested and the different methods of detection. Recent miRNA profiling studies have indicated that circulating miRNAs hold great potential as stable blood-based markers for human cancers^13,14^. The identification of novel, reliable, minimally invasive breast cancer biomarkers in body fluids, such as serum and plasma, would represent a significant development in the clinical management of this complex disease. Furthermore, previous data failed to validate altered expression of miR-301a in the blood of women with luminal A-like breast cancer, but the concept of a panel or profile of miRNAs for diagnostic purposes is a realistic approach, and to date, combined miRNAs have been reported with higher sensitivity, specificity and reproducibility^38^.

In conclusion, this study provides new insights into the role of miR-301a, not only in TNBC tumours but also in non-TNBC tumours. These results suggest that analysing miR-301a expression in the breast tissue biopsy specimen at the time of diagnosis could have the potential to identify patients who are at high risk for developing metastasis as well as patients who might be candidates for active surveillance. miR-301a may serve as a potential therapeutic target in patients with breast cancer.

## Materials and Methods

### Study population data

This is a retrospective study based on the data collected through the prospectively collected database from Fudan University Shanghai Cancer Centre (FDUSCC, Shanghai, China). A total of 380 female patients were involved in the study at the Department of Breast Surgery at FDUSCC from August 2001 to March 2006. Permission for this study was obtained from the Ethics Committee of FDUSCC, and informed consent was obtained from all participants or their legal guardians. We confirm that all methods were performed in accordance with the relevant guidelines and regulations. Clinical data and specimens from the study population were collected. All of them were pathologically diagnosed with breast invasive ductal carcinoma at FDUSCC. All patients were followed up regularly from the date of surgery to death. The last follow-up time was on March 2015, and the median follow-up time was 95.1 months (interquartile range [IQR]: 64.7–111.6 months). Apart from patient demographic characteristics, prognostic markers such as tumour size, tumour type, tumour grade, lymph node status, stage at diagnosis, oestrogen receptor (ER) and progesterone receptor (PR) status, and HER2 status were also analysed. Tumour staging was performed according to the American Joint Committee on Cancer criteria 6th edition (AJCC, http://www.cancerstaging.org). Baseline characteristics of the study cohort are shown in Table 1. Patients accepted adjuvant therapy following the guidelines of Chinese Anti-Cancer Association. Most of the patients (90.3%, 343/380) accepted chemotherapy, 28.4% of patients (108/380) had radiation therapy, and more than 90% of those who had ER-positive cancer received endocrine therapy.

### Tissue microarray (TMA) construction

The TMAs were constructed by the Department of Pathology at FDUSCC. Before cancer treatment, formalin-fixed and paraffin-embedded (FFPE) samples were collected from the patients. As previously described, representative tumour regions were identified using haematoxylin and eosin (HE) staining. In addition, tissue cylinders were then punched from these marked regions and transferred to recipient array blocks^39^. Duplicate cores (3 mm in diameter) were generated for comparison of the staining pattern in the same tumour.

### ***In situ*** hybridization for miR-301a

We used ISH to detect miR-301a expression with a digoxigenin(DIG)-labelled miRCURY LNA^TM^ probe on TMA sections. The DIG-labelled miRCURY LNA^TM^ detection probe for miR-301a was purchased from Exiqon (Vedbeak, Denmark) and the Enhanced Sensitive ISH Detection Kits were from Boster (Wuhan, China). The probe sequence was 5′-GCTTTGACAATACTATTGCACTG-3′. The detailed procedures of ISH were provided in our previous studies^31,32^.

### Staining evaluation

The staining of miR-301a was evaluated and independently and semi-quantitatively scored by two experienced pathologists. The observers were blinded to all patient clinical data and outcomes. The evaluation was accorded to the intensity of the staining and the score was the average of the two available cores. The staining was graded as follows: 0, no staining; 1, weak staining; 2, moderate staining; 3, strong staining. As in our previous studies^31,32^, a staining grade ≤ 2 was defined as miR-301a-negative staining, whereas only grade 3 staining was defined as positive staining. miR-301a was mainly localized in the cytoplasm of breast cancer cells.

### Statistical analysis

Descriptive statistics were employed to describe the epidemiological, clinical, and pathological diagnostic data. All statistical data were analysed using Statistical Package for the Social Sciences (SPSS) software (version 22.0; IBM Corporation). The main clinical outcomes in this study were disease-free survival (DFS) and overall survival (OS), which were calculated using the life-table method. DFS among patients with stage I to III diseases was defined as the time from the primary surgery to the first event of disease relapse or breast cancer-specific death. OS time was calculated from the time of diagnosis to the time of death from any cause. Patients without relapse or death and patients who were lost to follow up were censored at the last follow up. The relationships between miR-301a expression and clinicopathological parameters were examined using Pearson’s χ^2^ tests or Fisher’s exact tests when necessary. Survival curves for DFS and OS were plotted using the Kaplan-Meier method and were evaluated for the differences in survival using log-rank tests. The significance values of different variables with respect to survival (DFS/OS) were analysed using the univariate and multivariate Cox proportional hazards models. All P values were two-tailed and differences were considered statistically significant when *P* < 0.05. “Kaplan-Meier Plotter” is an online survival analysis tool that was used to assess the effect of 22,277 genes on breast cancer prognosis based on microarray data from 1,809 patients. Here, we utilized Kaplan-Meier Plotter to test miR-301a as a biomarker of breast cancer survival^33^.

### Data Availability

The datasets analysed during the current study are available from the corresponding author on reasonable request and the public data can be accessed online at www.kmplot.com/mirpower. On the main page, click “Start miRpower for breast cancer”, then type the name of the gene which you are interested under the textbox of “Select miRNA symbol”. Before generating Kaplan-Meier plot by clicking the button named “Draw Kaplan-Meier plot”, choose subtypes and cohorts to restrict analyses. All the details concerning how to use the online webtool was described in the paper published by Lanczky *et al*.^33^.
